# Exploring the Co-Crystallization of Kojic Acid with Silver(I), Copper(II), Zinc(II), and Gallium(III) for Potential Antibacterial Applications

**DOI:** 10.3390/molecules28031244

**Published:** 2023-01-27

**Authors:** Renren Sun, Lucia Casali, Raymond J. Turner, Dario Braga, Fabrizia Grepioni

**Affiliations:** 1 Dipartimento di Chimica “Giacomo Ciamician”, Università di Bologna, 40126 Bologna, Italy; 2 School of Chemical Engineering, Zhengzhou University, Zhengzou 450001, China; 3 Department of Biological Sciences, University of Calgary, Calgary, AB T2N 1N4, Canada

**Keywords:** antimicrobials, antimicrobial metals, silver, copper, gallium, zinc, co-crystallization, mechanochemistry

## Abstract

Co-crystallization of kojic acid (HKA) with silver(I), copper(II), zinc(II), or gallium(III) salts yielded three 1D coordination polymers and one 0D complex in which kojic acid was present as a neutral or anionic terminal or bridging ligand. All reactions were conducted mechanochemically via ball milling and manual grinding, or via slurry. All solids were fully characterized via single-crystal and/or powder X-ray diffraction. As kojic acid is a mild antimicrobial compound that is widely used in cosmetics, and the metal cations possess antibacterial properties, their combinations were tested for potential antibacterial applications. The minimal inhibition concentrations (MICs) and minimal biocidal concentrations (MBCs) for all compounds were measured against standard strains of the bacteria *P. aeruginosa*, *S. aureus*, and *E. coli*. All compounds exerted appreciable antimicrobial activity in the order of silver, zinc, copper, and gallium complexes.

## 1. Introduction

The search for new active antibacterial compounds is attracting increasing interest as the current clinical pipeline remains inadequate to meet the challenges of the growing emergence and spread of antimicrobial resistance [1]. According to the World Health Organization (WHO), the clinical benefit of newly approved products is limited compared to existing treatments, and most of the antimicrobials under clinical development are merely derivatives of molecules that are already known and used on the market, and bacteria have multiple resistance mechanisms that are well-established in the community [1]. Therefore, there is an urgent need to develop new antibacterial drugs. Historically, an increasing number of metal complexes have shown interesting biological and biomedical properties, such as the arsenic-containing organometallic complex for the treatment of syphilis (Salvarsan) [2], the mercuric-based antiseptic agent (Mercurochrome), and the gold complex agent for the treatment of rheumatoid arthritis (Auranofin) [3]. The metal complexes have a wide diversity of metals, ligand types, and geometries, which is an inbuilt advantage for accessing a highly undeveloped chemical space for drug development, especially for designing new antibacterial agents. 

**Scheme 1:**
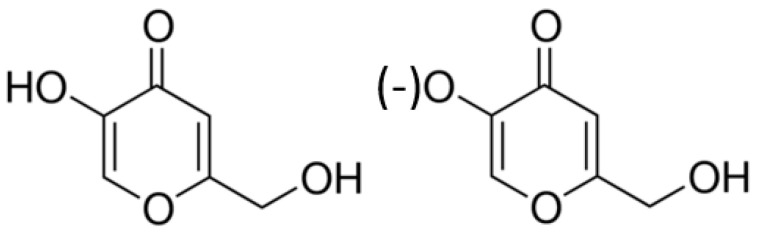
Kojic acid (HKA) and its monodeprotonated kojiate anion (KA^−^).

Kojic acid (HKA, see Scheme 1) is a common fungal metabolite that was first isolated from fungus. It can be used as a preservative for cosmetics, without one-time or cumulative irritation to the skin, and in a variety of foods, where it is added to prevent enzymatic browning [4]. It can strongly absorb ultraviolet rays and can be used alone or in combination with various sunscreen products and soaps [5]. It also can treat and prevent the formation of skin pigmentation, such as liver spots [6]. HKA also shows antibacterial properties, which make HKA and its derivatives widely explored as antibacterial agents for many types of bacteria [4,7]. 

Silver and its compounds have been widely used as antimicrobial agents [8], for example silver nitrate for the treatment of burn wounds [9] and silver sulfadiazine (Silvadene) as a broad-spectrum antibiotic for burn wounds [10]. The antimicrobial activities of zinc work via direct interactions with microbial membranes, leading to membrane instability and enhanced permeability [11] or via interactions with nucleic acids and the inactivation of respiratory enzymes [12]. In 1931, Levaditi et al. discovered the antibacterial properties of gallium by eradicating syphilis in rabbits and *Trypanosoma* in mice through the use of gallium tartrate [13]. Since then, various gallium compounds have advanced in preclinical and clinical investigations, such as those studying the potent suppressive activity of GaCl_3_ and Ga(III)-citrate on *P. aeruginosa* biofilm formation in vitro and in murine lung infection models [14]. Based on the toxic effects of free copper ions on both bacteria and fungi [15], the complexes conjoining organic molecules and copper were further studied in order to improve the antimicrobial activity; for example, copper complexes with sulfonamide ligands were synthesized for their antimicrobial properties [16], and copper complexes of 5-aminobenzofuran-2-carboxylate Schiff-base ligands have shown excellent antibacterial activity against *Mycobacteria tuberculosis* [17]. 

Our approach is based on the application of crystal engineering strategies to combine, hopefully in a synergistic way, the antimicrobial properties of organic molecules in the solid state with metal complexes to construct co-crystals and complexes. The preparation of co-crystals of known drugs, as well as the quest for new combinations of natural antibacterials, have been shown to be successful in a number of cases [18]. We have also contributed a series of papers in which we have described how these methods have been applied to inhibit enzymatic activity and to enhance the properties of known antibiotics [19,20].

In the present work, we explored the potential antibacterial properties of kojic acid in combination with metal cations by co-crystallizing kojic acid with silver(I), copper(II), zinc(II) and gallium(III) salts. Kojic acid, in combination with metal cations, has been used as a tyrosinase inhibitor [21,22] and as an anticancer agent [23]. The choice of copper and zinc was dictated by the fact that these are essential metal ions; therefore, they have a minor impact on the environment. Silver nitrate is a common antibacterial agent, and it is usually employed in healthcare to treat surfaces [9], but several bacteria have already developed resistance to silver ions [24,25,26]. Gallium salts are toxic, but, as mentioned above, they are already employed in specific treatments, and we are interested in exploring their possible antibacterial effects when used in stable combinations with organic ligands, as we have explored in our recent work [27,28]. 

**Scheme 2:**
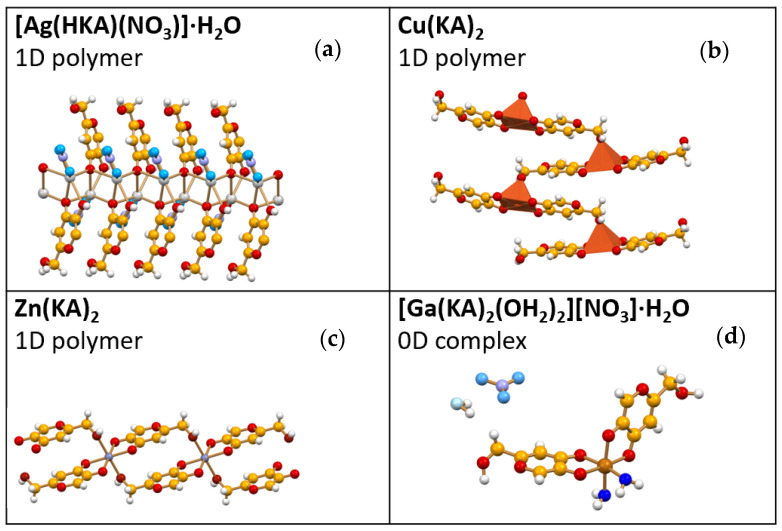
The products of the reactions of kojic acid with (**a**) silver(I), (**b**) copper(II), (**c**) zinc(II), and (**d**) gallium(III) salts.

In the following sections, we discuss the reaction of kojic acid with silver(I), copper(II), zinc(II), and gallium(III) salts that afforded the 1D polymers Ag(HKA)(NO_3_)∙H_2_O, Cu(KA)_2_, and Zn(KA)_2,_ and the 0D complex [Ga(KA)_2_(OH_2_)_2_][NO_3_]∙H_2_O, respectively, as shown in Scheme 2a–d. All products were characterized via X-ray diffraction, and their antimicrobial efficacy was tested against two Gram-negative organisms, *E. coli* and *P. aeruginosa*, and towards the Gram-positive strain *S. aureus*. 

## 2. Results and Discussion 

### 2.1. [Ag(HKA)(NO_3_)]∙H_2_O 

**Scheme 3:**
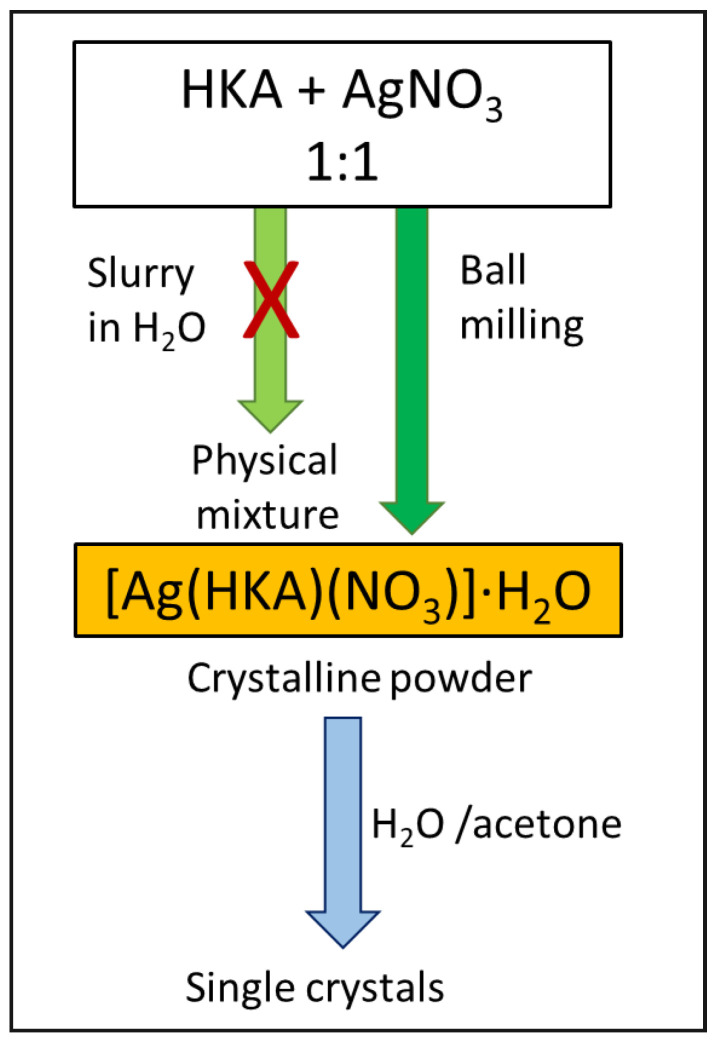
Details of the slurry, ball milling, and crystallization processes for the preparation of [Ag(HKA)(NO_3_)]∙H_2_O.

**Figure 1:**
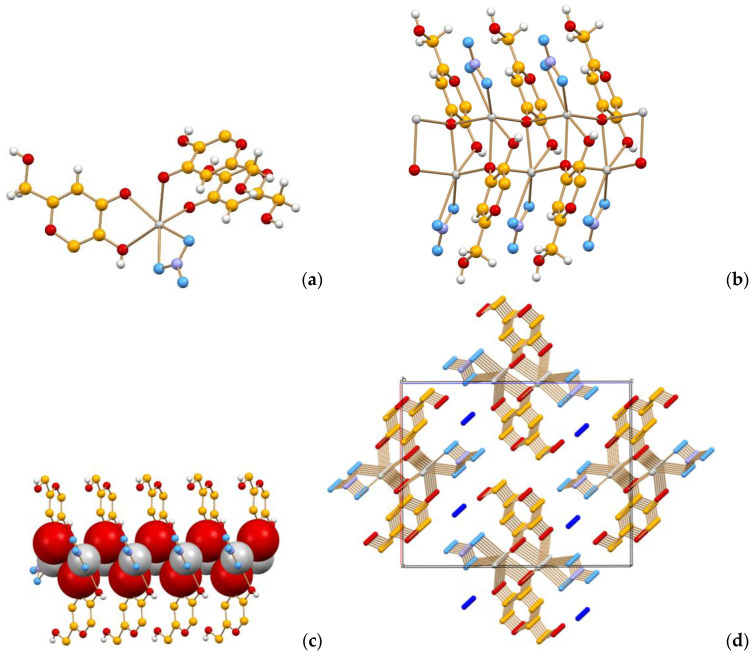
Coordination around the silver(I) ion in crystalline [Ag(HKA)(NO_3_)]∙H_2_O (**a**). The 1D ribbon, extending along the crystallographic *a*-axis, formed by the silver–oxygen backbone and the coordinated kojic acid molecules and nitrate ions (**b**,**c**) (H_CH_ atoms not shown for clarity). View of the packing down of the crystallographic *b*-axis, showing the water molecules (in blue) filling in the channel in between the 1D ribbons (**d**) (H atoms not shown for clarity). Water oxygen in blue, nitrate oxygens in cyan, and silver ions in light grey.

The results of ball milling, manual grinding, and crystallization from a solution of HKA and AgNO_3_ are represented in Scheme 3. While the slurry method resulted in a physical mixture of unreacted starting materials, ball milling and the subsequent crystallization in water/acetone resulted in the formation of the [Ag(HKA)(NO_3_)]∙H_2_O complex (see Figure 1).

The powder diffraction pattern for [Ag(HKA)(NO_3_)]∙H_2_O (see Figure S1 in the Supplementary Materials), calculated on the basis of the single-crystal structure, matches the experimental ones, confirming that the single-crystal structure is representative of both the crystallization and ball milling bulk products. Of all the complexes obtained in this work, this was the only one in which the kojic acid was still protonated, i.e., the complex can be described as a hydrated co-crystal of AgNO_3_ and kojic acid. The coordination around the silver cations in crystalline [Ag(HKA)(NO_3_)]∙H_2_O is shown in Figure 1a: each silver(I) is chelated by a kojic acid molecule, while a second and third HKA molecule act as terminal ligands via the carbonyl group (with Ag-O bond distances in the range 2.403–2.648(5) Å); the nitrate ion also coordinates the cation via two oxygen atoms (Ag-O_NO_3__ 2.408(4) and 2.604(5) Å). The HKA molecules bound to silver(I) via the carbonyl group are also chelating the neighbouring silver cations, as shown in Figure 1b, resulting in a 1D ribbon extending along the crystallographic *a*-axis (see the projections in Figure 1c,d), with the “Ag-O” backbone resembling an n-ladderane; the water molecules fill in the channels in between the 1D ribbons. 

### 2.2. Cu(KA)_2_

**Scheme 4:**
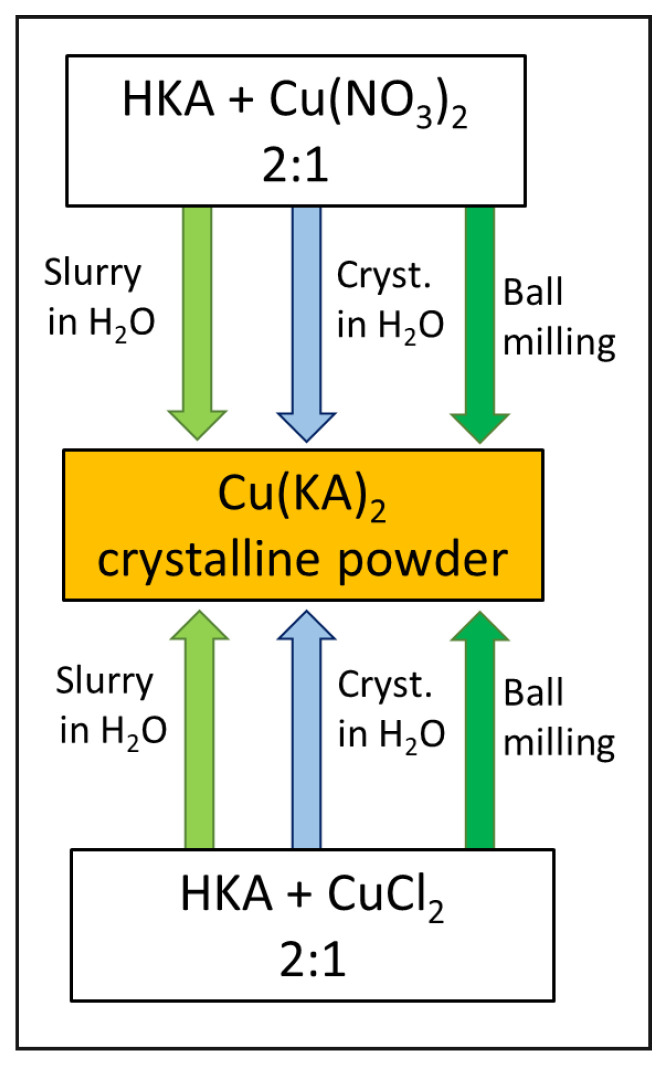
Details of the slurry, ball milling and crystallization processes for the preparation of Cu(KA)_2_.

Ball milling and slurrying of HKA and Cu(NO_3_)_2_ in a 2:1 stoichiometric ratio afforded the complex Cu(KA)_2_ (see Scheme 4). The powder diffraction patterns of the ball milling and slurrying products are shown in Figure S2 in the Supplementary Materials. Despite numerous attempts, no single crystal could be obtained due to poor solubility of the Cu(KA)_2_ crystalline powder in all of the solvents used (water, methanol, ethanol, acetone, chloroform, DMF, and DMSO). The same product was also obtained by reacting HKA with CuCl_2_ (see Scheme 4).

**Figure 2:**
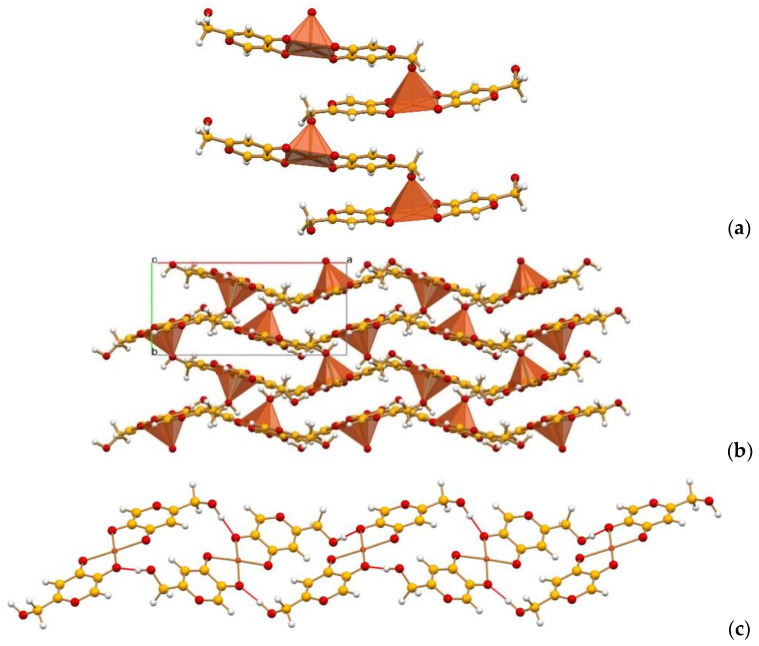
Square-pyramid polyhedra of oxygen atoms around the copper(II) cation in Cu(KA)_2_ (**a**); the 1D zig-zag chains are shown here (**b**); side-by-side, linked via O^−^⋯(H)O_CHOH_ hydrogen bonds (**c**). Copper ions are in dark orange.

The structure was thus solved from the powder data with the help of information derived from thermogravimetric analysis (TGA) (see Figures S4–S6 in the Supplementary Materials), which confirmed the absence of solvent in the crystal. In crystalline Cu(KA)_2_, the copper(II) cation is coordinated by five oxygen atoms: four belong to two kojate anions, acting as bidentate ligands and constituting the square base of a pyramidal polyhedron (see Figure 2a), while the fifth atom (the vertex) is represented by the O_CHOH_ group of a third kojate anion; this results in the formation of a 1D, zig-zag coordination polymer extending parallel to the crystallographic *b*-axis (Figure 2a). Neighbouring chains (Figure 2b) are linked via hydrogen bonds of the O^−^⋯(H)O_CHOH_ type (O⋯O distances 2.600–2.621(2) Å).

### 2.3. Zn(KA)_2_

**Scheme 5:**
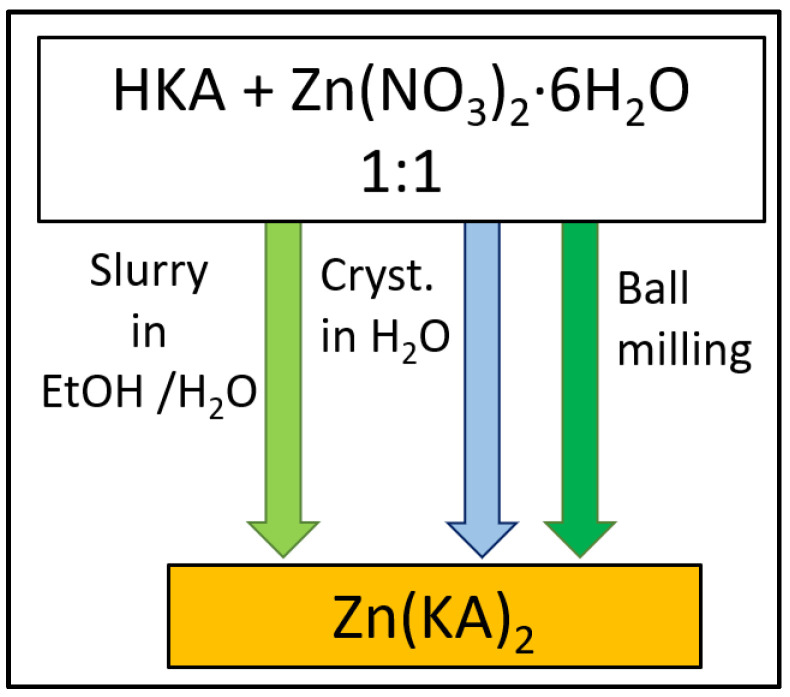
Details of the slurry, ball milling, and crystallization processes for the preparation of Zn(KA)_2_.

Analogously to Cu(KA)_2_, Zn(KA)_2_ was obtained via slurry, crystallization from solution, and ball milling HKA and Zn(NO_3_)_2_ in a 1:1 stoichiometric ratio (Scheme 5). 

**Figure 3:**
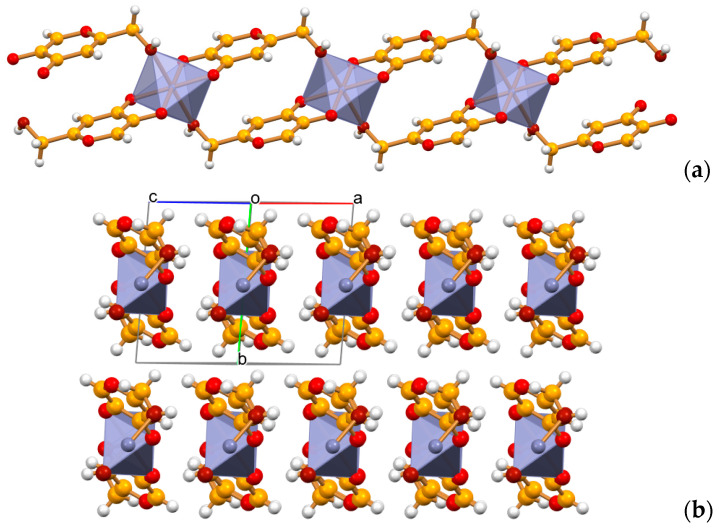
The 1D coordination polymer (**a**) extending along the (010) direction in crystalline Zn(KA)_2_; note how the six-membered rings are π-stacked within the chain at a distance of ca. 3.2 Å. 1D chains are arranged parallel to each other in the crystal (**b**). Zinc(II) cations are blue-grey.

The powder diffraction patterns for the bulk products and the one calculated on the basis of single-crystal data are compared in Figure S3 in the Supplementary Materials. While a period of 60 min of ball milling (see also Section 3) was not sufficient for the reaction to occur quantitatively, peaks of unreacted material were almost negligible in the product of the slurry process. The crystal structure of Zn(KA)_2_ is shown in Figure 3. Two kojiate anions bridge two Zn(II) as bidentate (Zn-O_COO^−^_ distances: 2.05(2) and 2.06(2) Å) and monodentate (Zn-O_OH_ distance: 2.21(2) Å) ligands, thus forming 1D polymers extending in the [101] crystallographic direction and parallel to each other in the crystal (Figure 3a,b); the zinc cations are located on centres of symmetry within octahedra of oxygen atoms (see Figure 3a). The six-membered rings are π-stacked within the 1D polymer, with a distance between the two mean planes of ca. 3.2 Å. 

### 2.4. [Ga(KA)_2_(OH_2_)_2_][NO_3_]∙H_2_O

**Scheme 6:**
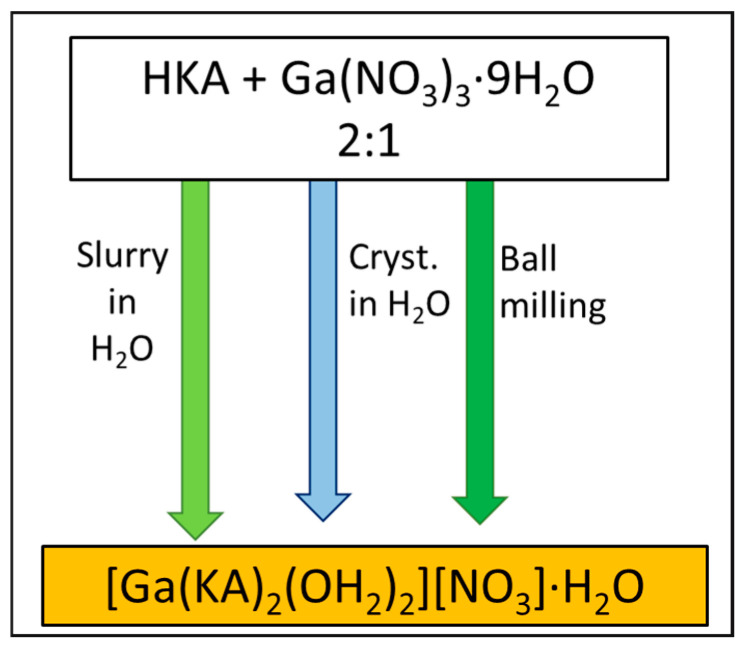
Details of the slurry, ball milling and crystallization processes for the preparation of [Ga(KA)_2_(OH_2_)_2_][NO_3_]∙H_2_O.

Ball milling, slurrying, and crystallization from the solution of HKA with Ga(NO_3_)_3_ in a 2:1 stoichiometric ratio resulted in the metal complex [Ga(KA)_2_(OH_2_)_2_][NO_3_]∙H_2_O (see Scheme 6).

**Figure 4:**
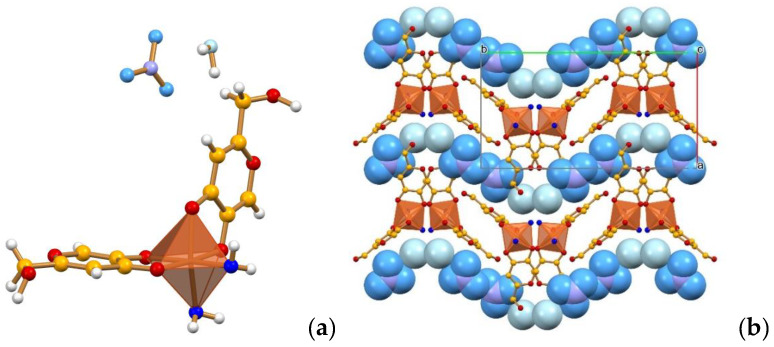
Crystalline [Ga(KA)_2_(OH_2_)_2_][NO_3_]∙H_2_O: (**a**) Octahedral coordination around the Ga^3+^ cation, and (**b**) intercalation of nitrate anions/water molecules between wavy layers of cations extending along the *b*-axis direction. (H atoms not shown for clarity). Water oxygens are in blue (coordination sphere) and light blue (external); nitrate oxygens are in cyan, and nitrogens are in violet.

The powder diffraction pattern for the complex, calculated on the basis of single crystal data, matches the one measured on the bulk products (see Figure S4). Differently from what observed for the silver nitrate co-crystal, when gallium nitrate is reacted with HKA the kojic acid is deprotonated, and acts as a bidentate ligand toward the gallium cation. Two KA^−^ anions coordinated the gallium cation (see Figure 4a), and two water molecules completed the octahedral coordination sphere while the nitrate anion was at hydrogen bonding distance from the water molecule that did not belong to the coordination sphere and from the -OH group on the KA^−^ anion. Wave layers of cations can be observed extending along the *b*-axis direction, with nitrate anions and the third water molecule occupying the space among the layers (Figure 4b).

### 2.5. Antimicrobial Assays

**Figure 5:**
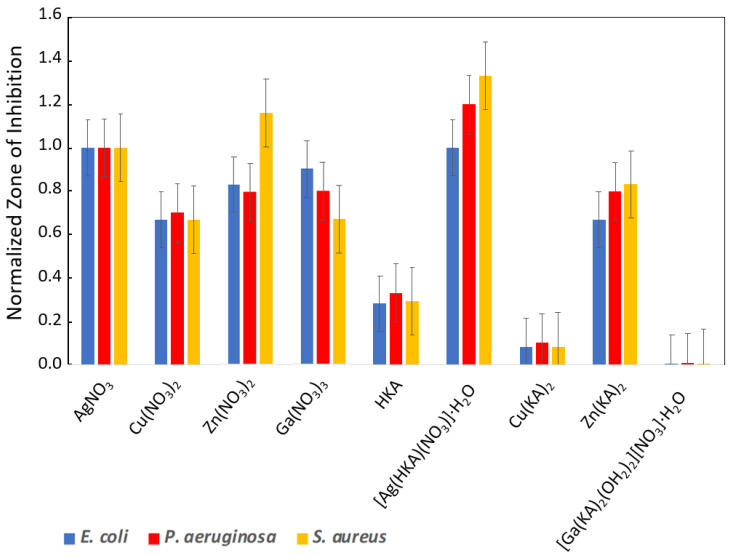
Kirby–Bauer disk-diffusion assay on LB agar plates. Distance zones were normalized to that of AgNO_3_ as a value of 1.0. The taller the bar, the more antimicrobial the compound is. Standard errors are presented from 3–5 trials.

Three pathogen-indicator strains were used to assess antimicrobial efficacy. A classical disk-diffusion assay was performed to assess whether any antimicrobial activity existed. This type of assay also evaluates some physical characteristics of the materials. For this type of assay, the compound/crystal is bound/trapped/adsorbed to a filter paper and its polymer strands. This filter disk is then placed on agar bacterial growth media (effectively, a type of hydrogel). The compound must then be released from the filter paper and diffuse through the agar, leading to a concentration gradient away from the disk. The relative size of the zone of no growth around the disk is then evaluated, and the resulting data are shown in Figure 5. One can observe that, as expected, AgNO_3_ is an effective antimicrobial against the two Gram-negative organisms (*E. coli* and *P. aeruginosa*), while the best activity towards the Gram-positive strain *S. aureus* comes from Zn(NO_3_)_2_. The copper and gallium salts also showed good antimicrobial activity, as the metal ions diffused readily through the agar and are known antimicrobial metals [29]. The kojic acid on its own showed about one-third the antimicrobial efficacy of AgNO_3_, validating previous reports of antimicrobial activity. As a comparator, the beta-lactam antibiotic ampicillin shows good antimicrobial activity relative to AgNO_3_ with a normalized zone of inhibition (NZ) of 1.7 for *E. coli*. Ampicillin is one-half as effective (NZ = 0.5) against *P. aeruginosa*, but twice as effective against *S. aureus* (NZ = 2.2).

As for the metal complexes, we observed diverse outcomes. Mechanistically, we do not know if these complexes released from the filter paper equivalently, nor can we be certain about their diffusion properties through the hydrogel agar media. Regardless, with this assay, we were able to see that both [Ag(HKA)(NO_3_)]∙H_2_O and Zn(KA)_2_ displayed antimicrobial activity towards all three tested bacterial strains.

Given the range of results of the metal–kojic acid complexes in the disk-diffusion assay, we also performed serial dilution experiments in broth culture to obtain the minimal inhibitory concentrations (MICs) and minimal biocidal concentrations (MBCs) in order to evaluate the bacteriostatic and bactericidal properties, respectively (see Table 1 and Table 2). In this case, the metal complexes do not have to dissociate from a filter paper matrix nor diffuse through a hydrogel. The bacteria and the complexes can fully intermingle in the broth medium. Similar to the Kirby–Bauer disk-diffusion assay, for both the MIC and the MBC, the smaller the value, the better the antimicrobial activity, and as typically observed, the MBC is usually 1–4 dilution steps away from the MIC as higher concentrations are required to kill rather than to just inhibit.

These broth experiments reflect the strong antimicrobial efficacy of each of the metal salts and the different efficacies of the different strains [30,31]. We also saw that kojic acid had intermediate MICs towards all three organisms with equivalent MBCs, except there was no biocidal activity against *S. aureus*, which may reflect a difference in the penetration of the cell wall of the Gram-positive bacterium. We saw strong MIC and MBC values for the silver–kojic acid complex, while the other metal complexes, although showing intermediate bacteriostatic MIC values, were unable to kill any of the strains. 

Overall, our data suggests that the silver– and zinc–kojic acid complexes were able to release their antimicrobial components in both agar (hydrogel) and broth (solution) conditions. The copper complex showed curious results as its antimicrobial activity via disk-diffusion was poor, but it was able to work against the Gram-positive bacteria in the broth culture. Perhaps in an aqueous medium, the crystals can interact more easily with the complex cell wall comprised of peptidoglycans and teichoic acid polymers, which leads to an arrested cellular division. We also saw that, although the [Ga(KA)_2_(OH_2_)_2_][NO_3_]∙H_2_O co-crystal did not display antimicrobial activity via disk diffusion, it demonstrated quite good bacteriostatic properties via MIC (see Table 1).

## 3. Materials and Methods

### 3.1. Materials 

Kojic acid (purity > 98.0%) was purchased from TCI. AgNO_3_ (purity > 99.0%), Zn(NO_3_)_2_·6H_2_O (purity > 98.0%), Cu(NO_3_)_2_·3H_2_O (purity > 99.0%), and Ga(NO_3_)_3_·9H_2_O (purity > 99.9%) were purchased from Sigma-Aldrich (Burlington, MA, USA).

### 3.2. Mechanochemical Synthesis

[Ag(HKA)(NO_3_)]∙H_2_O was obtained by ball milling equimolar quantities of AgNO_3_ (0.5 mmol) and HKA (0.5 mmol) in a 5 mL agate jar with two 3 mm agate balls in the presence of two drops (100 μL) of water and for 60 min in a Retsch MM200 ball miller operated at a frequency of 20 Hz. 

Zn(KA)_2_ and Cu(KA)_2_ were obtained by ball milling Zn(NO_3_)_2_·6H_2_O and Cu(NO_3_)_2_·3H_2_O (0.3 mmol in both cases) and HKA (0.6 mmol) in a 1:2 stoichiometric ratio in 5 mL agate jars with two 3 mm agate balls in the presence of one drop (50 μL) of water, for 60 min in a Retsch MM200 ball miller operated at a frequency of 20 Hz. 

[Ga(KA)_2_(OH_2_)_2_][NO_3_]∙H_2_O was obtained by ball milling Ga(NO_3_)_3_·9H_2_O (0.3 mmol) and HKA (0.6 mmol) in a 1:2 stoichiometric ratio in a 5 mL agate jar with two 3 mm agate balls in the presence of one drop of water (50 μL), for 20 min in a Retsch MM200 ball miller operated at a frequency of 20 Hz. 

In all cases, if different stoichiometric ratios were used, the same products were obtained and excess reagent was found unreacted in the resulting crystalline powders.

### 3.3. Slurry Syntheses

[Ga(KA)_2_(OH_2_)_2_][NO_3_]∙H_2_O, Zn(KA)_2_ and Cu(KA)_2_ crystalline powders were also obtained by slurrying 1:2 stoichiometric ratios of metal compounds (0.5 mmol) and HKA (1.0 mmol) in 5 mL of water. 

### 3.4. Solution Syntheses

Single crystals of [Ag(HKA)(NO_3_)]∙H_2_O and [Ga(KA)_2_(OH_2_)_2_][NO_3_]∙H_2_O were obtained by the slow solvent evaporation at room temperature of an aqueous solution (5 mL) containing 0.5 mmol of the silver compounds and 0.3 mmol of the gallium compounds in a 1:1 and a 1:2 stoichiometric ratio with kojic acid, respectively. Zn(KA)_2_ single crystals were obtained via the solvent evaporation of a solution obtained by dissolving the ball milling product in the minimum amount of water and leaving the solution in the open air at ambient conditions.

### 3.5. Thermogravimetric Analysis

In this work, a TGA instrument (PerkinElmer TGA7) was employed to determine the behaviour of Cu(KA)_2_, which could be instrumental to the structure solution from powder data. Cu(KA)_2_ (1.936 mg) was placed in an aluminium pan and measured at the 305–725 K temperature range at a heating rate of 5 °C·min^−1^ under an N_2_ atmosphere.

### 3.6. Single-Crystal X-ray Diffraction

Single-crystal X-ray diffraction data were collected with an Oxford Diffraction Xcalibur equipped with a graphite monochromator and a CCD detector. Mo-Kα radiation (λ = 0.71073 Å) was used. Single-crystal X-ray diffraction data for Zn(KA)_2_ and [Ga(KA)_2_(OH_2_)_2_][NO_3_]∙H_2_O was collected at room temperature, while diffraction data for [Ag(HKA)(NO_3_)]∙H_2_O was collected at a low temperature (223 K). Table S1 in the Supplementary Materials reports the crystallographic details for all of the complexes. The structuresresolution and refinement by the least-squares method against F^2^ were carried out using SHELXT [32] and SHELXL [33], respectively, with the Olex2 [34] interface. Nonhydrogen atoms were refined anisotropically. Hydrogen atoms were added in calculated positions. The software Mercury [35] was used to calculate the powder patterns basing on single-crystal data and for molecular graphics. The CCDC numbers 2226706-2226709 contain the supplementary crystallographic data for this paper. These data can be obtained free of charge via http://www.ccdc.cam.ac.uk/conts/retrieving.html (accessed on 15 December 2022) (or from the CCDC, Cambridge CB2 1EZ, England; Fax: +44 1223 336033; E-mail: deposit@ccdc.cam.ac.uk).

### 3.7. X-ray Diffraction from Powder

For phase identification, powder X-ray diffraction (PXRD) data was collected on a PANalytical X’Pert Pro automated diffractometer equipped with an X’Celerator detector in Bragg–Brentano geometry, using Cu-Kα radiation (λ = 1.5418 Å) without a monochromator, in the 2θ range 5–40° (step size: 0.033°; time/step: 40 s; Soller slit: 0.04 rad; antiscatter slit: 1/2; divergence slit: 1/4; 40 mA × 40 kV). For structure solution purposes, in the case of Cu(KA)_2_, PXRD patterns were collected on a PANalytical X’Pert Pro automated diffractometer with transmission geometry equipped with a focusing mirror and a PIXcel detector, using Cu-Kα radiation (λ = 1.5418 Å) without a monochromator, in the 2θ range 3–70° (step size 0.0130°, time/step 200 s, Soller slit: 0,04 rad; antiscatter slit: 1/2; divergence slit: 1/4; 40 kV × 40 mA). To improve the data quality, three repetitions were performed, and the results of the three scans were combined for the final result.

### 3.8. Structural Characterization from Powder Data 

The powder diffraction data for Cu(KA)_2_ was analysed with PANalytical X’Pert HighScore Plus software. Structure solution was performed with EXPO2014 [36] by simulated annealing in the space group P21 using two KA molecules and one Cu atom. The simulated annealing trials were run 10 times. The best solution was chosen for a subsequent Rietveld refinement using TOPAS4.1 software [37]. 

### 3.9. Antimicrobial Analysis

The antimicrobial susceptibility of the metal salts and the kojic acid co-crystals were performed in two different ways. As in our previous works [20,27,38], we performed the initial antimicrobial assessment with the Kirby–Bauer disk-diffusion assaywith minor modifications from the standard testing protocol [39]. The following standard strains were used in this study: *Pseudomonas aeruginosa* ATCC27853, *Staphylococcus aureus* ATCC25923, and *Escherichia coli* ATCC25922. All culturing was performed in lysogeny broth (LB) prepared in distilled water with 10 g/L NaCl (VWR International Co., Mississauga, ON, Canada), 5 g/L yeast extract (EMD Chemicals Inc., Darmstadt, Germany), and 10 g/L tryptone (VWR Chemicals LLC, Solon, OH, USA). Agar medium for the disk-diffusion experiments was made by adding 15 g/L bacteriological agar (VWR International LLC, Solon, OH, USA) to the LB. In an aseptic environment, 200 μL of overnight culture of *S. aureus*, *P. aeruginosa*, or *E. coli* was spread on the LB agar plates (two plates per organism for two technical replicates) and left to dry at room temperature for 1 h.

Stocks of 24 mg/mL stock solutions of single components of the metal nitrate salts and suspensions/slurries of the target co-crystal compounds were prepared from powder using the LB media. Four blank antimicrobial disks (Oxoid Ltd., Basingstoke, UK) were placed into a vial with 300 μL of compound stocks and left to soak for 30 min with mixing every 10 min by inversion of the vial. Then, the disks were transferred to the plates, and the plates incubated at 37 °C for 24 h. Every plate had an AgNO_3_ disk as a control. This allowed for plate-to-plate and trial variations to be removed through normalization to the zone of inhibition for AgNO_3_. The zone of growth inhibition was measured by a ruler and/or photographed and enlarged in order to quantify smaller zones more easily. Experiments were repeated with 3–5 different biological trials. We note that there may have been differing amounts of co-crystals adsorbed onto each disk within a vial; however, little variation was seen between trials. An additional unknown is whether all of the different co-crystals adsorbed to the filter disks equivalently, or the release of the co-crystals from the disks was equivalent. Yet, the assay is still valid for the interpretation efficacy of antimicrobial applications, such as in a wound dressing.

The second approach was to use the microtiter plate serial two-fold dilutions in LB broth media to obtain the minimum inhibitory concentration (MIC) [39]. Using a standard microtiter plate, each experiment had 8 sterility control wells (150 µL of medium), 8 growth control wells (75 µL of medium), and 2 replicates of dilution series for the different compounds (for a total of 80 wells). A quantity of 75 µL of medium, of which was added 75 µL of the mixture in first well, was then mixed by pipetting up and down a few times. Then, 75 µL was transferred to the next well for a one-half dilution. A 1% inoculant in the LB was prepared from an overnight saturated culture of each of the 3 strains. Inoculation of the microtiter plate was obtained by transferring 75 µL of the 1% culture to each well and mixing for a final volume of 150 µL. Thus, the first-row concentration was diluted from 25 mg/mL to 12.5 mg/mL, and all wells were diluted again by 50%, giving a dilution series ranging from 6.25 to 0.049 mg/mL. The plates were incubated for 24 h at 37 °C and shaken at 150 rpm in a humidity chamber to protect against evaporation. The cultured plates were analysed visually and by optical density at 600 nm, and the MICs were identified as the concentration of the compound in the first well that had no increase in turbidity, adjacent to the well that had such changes present. From this microtiter plate, the minimal biocidal concentration (MBC) was assessed by using a pined replica plater to transfer a 3–5 µL spot to an LB plate and to a fresh microtiter plate with 150 µL per well to assess growth recovery after a further 24 h of incubation at 37 °C. Growth was scored as +/− for each dilution, and the last dilution with no growth recovery was defined as the MBC. Experiments were performed with 3 biological replicates. In Table 1 and Table 2, a value of >6.25 indicates the upper limit of the assay, and thus no MBC end point was reached.

## 4. Conclusions 

In this paper, we have reported on the synthesis, characterization, and evaluation of the antimicrobial activity of a series of novel coordination compounds of the mild antibiotic compounds of kojic acid with metal salts of silver, zinc, copper, and gallium. The new compounds, namely [Ag(HKA)(NO_3_)]∙H_2_O, Zn(KA)_2_, Cu(KA)_2_, and Ga(KA)_2_(OH_2_)_2_][NO_3_]∙H_2_O, were prepared by mechanochemical, slurry, and solution methods and structurally investigated via single-crystal and powder diffraction. The structures of the silver, zinc, and copper products are 1D coordination polymers, whereby the kojic acid or its deprotonated kojiate anion act as a bridge between the metal centres. The gallium(III) complex formed, instead, a 0D compound bearing two kojiate anions and two water molecules in the coordination sphere. 

As the individual components are antimicrobial, we evaluated whether their activity was effected in the co-crystal form, and thus all compounds were tested against the standard pathogen strains of the bacteria *P. aeruginosa*, *S. aureus*, and *E. coli* via disk-diffusion assay measurement. [Ag(HKA)(NO_3_)]∙H_2_O was the most effective compound, while [Ga(KA)_2_(OH_2_)_2_][NO_3_]∙H_2_O was the least effective. Indeed, the silver and zinc kojic acid 1D coordination polymers were able to release their antimicrobial components in both agar and broth conditions, while the copper complex appeared to work against the Gram-positive bacteria in the broth culture. As far as the gallium 0D compound is concerned, while there was no antimicrobial activity via disk-diffusion, a good level of bacteriostatic activity was shown by the MIC. 

Overall, our findings have met the objective of this work, mentioned in the introduction, namely the quest for potentially useful, new antimicrobials to meet the growing challenge posed by the increase in antimicrobial resistance by pathogens due to the overuse of antibiotics. We believe that the combination of the antimicrobial properties of organic molecules with those of the metal atoms in the co-crystals and coordination compounds could represent steps forward in this direction. In this same line of work, we have recently shown that the antimicrobial compound proflavine can be co-crystallized with metal salts of silver, zinc, and gallium, yielding an increase in the minimal inhibition concentration of the drug activity towards bacteria [27,38]. However, these are only initial steps as considerable thought is required to understand the mechanisms of action of co-crystals and coordination polymers against bacteria. These studies, which require further, more specialized, and collaborative investigations, are under way.

## Figures and Tables

**Table 1:** Minimum inhibitory concentrations (MICs) in LB broth media (mg/mL).

	*E. coli*	*P. aeruginosa*	*S. aureus*
AgNO_3_	<0.05	<0.05	<0.05
Cu(NO_3_)_2_	0.78	1.50	1.50
Zn(NO_3_)_2_	6.25	3.15	1.50
Ga(NO_3_)_3_	1.50	0.40	0.78
HKA	6.25	3.10	1.50
[Ag(HKA)·NO_3_]·H_2_O	<0.05	<0.05	<0.05
Cu(KA)_2_	6.25	6.25	1.56
Zn(KA)_2_	3.00	1.50	3.00
[Ga(KA)_2_(OH_2_)_2_][NO_3_]·H_2_O	3.00	3.10	3.00

**Table 2:** Minimum biocidal concentrations (MBCs) as evaluated by regrowth in both broth and agar media (mg/mL).

	*E. coli*	*P. aeruginosa*	*S. aureus*
AgNO_3_	0.10	0.05	0.10
Cu(NO_3_)_2_	3.13	1.56	3.13
Zn(NO_3_)_2_	3.13	3.13	6.25
Ga(NO_3_)_3_	3.13	3.13	3.13
HKA	3.13	3.13	>6.25
[Ag(HKA)·NO_3_]·H_2_O	0.10	0.10	0.98
Cu(KA)_2_	>6.25	>6.25	>6.25
Zn(KA)_2_	>6.25	>6.25	>6.25
[Ga(KA)_2_(OH_2_)_2_][NO_3_]·H_2_O	>6.25	6.25	>6.25

## Data Availability

Experimental details can be obtained from the Supplementary Materials. CCDC numbers 2226706-2226709 contain the supplementary crystallographic data for this paper. These data can be obtained free of charge via http://www.ccdc.cam.ac.uk/conts/retrieving.html (accessed on 15 December 2022) (or from the CCDC, 12 Union Road, Cambridge CB2 1EZ, UK; Fax: +44 1223 336033; E-mail: deposit@ccdc.cam.ac.uk).

## Supplementary Materials

The following supporting information can be downloaded at: https://www.mdpi.com/article/10.3390/molecules28031244/s1, Table S1: Crystal data and details of measurements; Figures S1–S4: Powder X-ray diffraction patterns; Figure S5: Pawley refinement for Cu(KA)_2_); Figure S6: TGA for Cu(KA)_2_.

## Sample Availability

No samples of the compounds are available from the authors.

## References

[1] (2019). 2019 Antibacterial Agents in Clinical Development: An Analysis of the Antibacterial Clinical Development Pipeline.

[2] Lloyd N.C., Morgan H.W., Nicholson B.K., Ronimus R.S. (2005). The composition of Ehrlich’s salvarsan: Resolution of a century-old debate. Angew. Chem. Int. Ed..

[3] Ghosh S. (2019). Cisplatin: The first metal based anticancer drug. Bioorg. Chem..

[4] Burdock G.A., Soni M.G., Carabin I.G. (2001). Evaluation of health aspects of kojic acid in food. Regul. Toxicol. Pharmacol..

[5] Saeedi M., Eslamifar M., Khezri K. (2019). Kojic acid applications in cosmetic and pharmaceutical preparations. Biomed. Pharmacother..

[6] Miyabe C., Dong Y., Wakamatsu K., Ito S., Kawakami T. (2020). Kojic acid alters pheomelanin content in human induced pluripotent stem cell-derived melanocytes. J. Dermatol..

[7] Saraei M., Zarrini G., Esmati M., Ahmadzadeh L. (2017). Novel functionalized monomers based on kojic acid: Snythesis, characterization, polymerization and evalution of antimicrobial activity. Des. Monomers Polym..

[8] Vishwanath N., Whitaker C., Allu S., Clippert D., Jouffroy E., Hong J., Stone B., Connolly W., Barrett C.C., Antoci V. (2022). Silver as an antibiotic-independent antimicrobial: Review of current formulations and clinical relevance. Surg. Infect..

[9] Alexander J.W. (2009). History of the medical use of silver. Surg. Infect..

[10] Ballin J.C. (1974). Evaluation of a new topical agent for burn therapy: Silver sulfadiazine (Silvadene). JAMA.

[11] Fang M., Chen J.-H., Xu X.-L., Yang P.-H., Hildebrand H.F. (2006). Antibacterial activities of inorganic agents on six bacteria associated with oral infections by two susceptibility tests. Int. J. Antimicrob. Agents.

[12] Yamgar R.S., Nivid Y., Nalawade S., Mandewale M., Atram R., Sawant S.S. (2014). Novel Zinc(II) complexes of heterocyclic ligands as antimicrobial agents: Synthesis, characterisation, and antimicrobial studies. Bioinorg. Chem. Appl..

[13] Levaditi C., Bardet J., Tchakirian A., Vaisman A. (1931). Le gallium, propriétés thérapeutiques dans la syphilis et les trypanosomiases expérimentales. CR Hebd. Seances Acad. Sci. Ser. D Sci. Na.

[14] Kaneko Y., Thoendel M., Olakanmi O., Britigan B.E., Singh P.K. (2007). The transition metal gallium disrupts *Pseudomonas aeruginosa* iron metabolism and has antimicrobial and antibiofilm activity. J. Clin. Investig..

[15] Sweetman S.C. (2014). Martindale: The complete drug reference. 38th ed.. Aust. Prescr..

[16] Kremer E., Facchin G., Estévez E., Alborés P., Baran E., Ellena J., Torre M. (2006). Copper complexes with heterocyclic sulfonamides: Synthesis, spectroscopic characterization, microbiological and SOD-like activities: Crystal structure of [Cu(sulfisoxazole)_2_(H_2_O)_4_]·2H_2_O. J. Inorg. Biochem..

[17] Nazirkar B., Mandewale M., Yamgar R. (2019). Synthesis, characterization and antibacterial activity of Cu(II) and Zn(II) complexes of 5-aminobenzofuran-2-carboxylate Schiff base ligands. J. Taibah Univ. Sci..

[18] Bolla G., Sarma B., Nangia A.K. (2022). Crystal Engineering of Pharmaceutical Cocrystals in the Discovery and Development of Improved Drugs. Chem. Rev..

[19] Shemchuk O., d’Agostino S., Fiore C., Sambri V., Zannoli S., Grepioni F., Braga D. (2020). Natural antimicrobials meet a synthetic antibiotic: Carvacrol/thymol and ciprofloxacin cocrystals as a promising solid-state route to activity enhancement. Cryst. Growth Des..

[20] Fiore C., Baraghini A., Shemchuk O., Sambri V., Morotti M., Grepioni F., Braga D. (2022). Inhibition of the Antibiotic Activity of Cephalosporines by Co-Crystallization with Thymol. Cryst. Growth Des..

[21] Ashooriha M., Khoshneviszadeh M., Khoshneviszadeh M., Moradi S.E., Rafiei A., Kardan M., Emami S. (2019). 1,2,3-Triazole-based kojic acid analogs as potent tyrosinase inhibitors: Design, synthesis and biological evaluation. Bioorg. Chem..

[22] Ashooriha M., Khoshneviszadeh M., Khoshneviszadeh M., Rafiei A., Kardan M., Yazdian-Robati R., Emami S. (2020). Kojic acid–natural product conjugates as mushroom tyrosinase inhibitors. Eur. J. Med. Chem..

[23] Lachowicz J.I., Mateddu A., Coni P., Caltagirone C., Murgia S., Gibson D., Dalla Torre G., Lopez X., Meloni F., Pichiri G. (2022). Study of the DNA binding mechanism and in vitro activity against cancer cells of Iron(III) and Aluminium(III) kojic acid derivative complexes. Dalton Trans..

[24] Hosny A.E.-D.M., Rasmy S.A., Aboul-Magd D.S., Kashef M.T., El-Bazza Z.E. (2019). The increasing threat of silver-resistance in clinical isolates from wounds and burns. Infect. Drug Resist..

[25] Panáček A., Kvítek L., Smékalová M., Večeřová R., Kolář M., Röderová M., Dyčka F., Šebela M., Prucek R., Tomanec O. (2018). Bacterial resistance to silver nanoparticles and how to overcome it. Nat. Nanotechnol..

[26] Muller M., Merrett N.D. (2014). Pyocyanin production by Pseudomonas aeruginosa confers resistance to ionic silver. Antimicrob. Agents Chemother..

[27] Guerrini M., d’Agostino S., Grepioni F., Braga D., Lekhan A., Turner R.J. (2022). Antimicrobial activity of supramolecular salts of Gallium(III) and proflavine and the intriguing case of a trioxalate complex. Sci. Rep..

[28] Lekhan A., Fiore C., Shemchuk O., Grepioni F., Braga D., Turner R.J. (2022). Comparison of Antimicrobial and Antibiofilm Activity of Proflavine Co-crystallized with Silver, Copper, Zinc, and Gallium Salts. ACS Appl. Bio. Mater..

[29] Lemire J.A., Harrison J.J., Turner R.J.J.N.R.M. (2013). Antimicrobial activity of metals: Mechanisms, molecular targets and applications. Nat. Rev. Microbiol..

[30] Gugala N., Lemire J.A., Turner R.J. (2017). The efficacy of different anti-microbial metals at preventing the formation of, and eradicating bacterial biofilms of pathogenic indicator strains. J. Antibiot..

[31] Gugala N., Vu D., Parkins M.D., Turner R.J. (2019). Specificity in the susceptibilities of *Escherichia coli*, *Pseudomonas aeruginosa* and *Staphylococcus aureus* clinical isolates to six metal antimicrobials. Antibiotics.

[32] Sheldrick G.M. (2015). SHELXT–Integrated space-group and crystal-structure determination. Acta Crystallogr. Sect. A Found. Adv..

[33] Sheldrick G. (2013). XS.

[34] Dolomanov O.V., Bourhis L.J., Gildea R.J., Howard J.A., Puschmann H. (2009). OLEX2: A complete structure solution, refinement and analysis program. J. Appl. Crystallogr..

[35] Macrae C.F., Edgington P.R., McCabe P., Pidcock E., Shields G.P., Taylor R., Towler M., Streek J. (2006). Mercury: Visualization and analysis of crystal structures. J. Appl. Crystallogr..

[36] Altomare A., Cuocci C., Giacovazzo C., Moliterni A., Rizzi R., Corriero N., Falcicchio A. (2013). EXPO2013: A kit of tools for phasing crystal structures from powder data. J. Appl. Crystallogr..

[37] Cohelo A. (2007). TOPAS-Academic.

[38] Fiore C., Shemchuk O., Grepioni F., Turner R.J., Braga D. (2021). Proflavine and zinc chloride “team chemistry”: Combining antibacterial agents via solid-state interaction. CrystEngComm.

[39] Balouiri M., Sadiki M., Ibnsouda S.K. (2016). Methods for in vitro evaluating antimicrobial activity: A review. J. Pharm. Anal..

