# Supplementary material for: Exploring the Co-Crystallization of Kojic Acid with Silver(I), Copper(II), Zinc(II), and Gallium(III) for Potential Antibacterial Applications
Source: Molecules. 2023 Jan 27;28(3):1244. doi: 10.3390/molecules28031244 (PMC9920434; doi:10.3390/molecules28031244)
Supplement: Supplementary file 1 [file molecules-28-01244-s001.zip › molecules-2141649-supplementary.pdf]

Supporting Information

(5 pages)

Exploring the co-crystallization of kojic acid with copper(II), silver (I), zinc(II) and gallium(III) for potential antibacterial applications.

Renren Sun,[a,b] Lucia Casali,[a] Raymond J. Turner,[c,*] Dario Braga[a] and Fabrizia Grepioni[a,*]

a. Dipartimento di Chimica "Giacomo Ciamician", Università di Bologna, Via Selmi 2, 40126 Bologna, Italy.
b. School of Chemical Engineering, Zhengzhou University, 450001, Zhengzou, Henan Province, The People's Republic of China.
c. Department of Biological Sciences, University of Calgary, Calgary, Alberta, Canada, T2N 1N4.

Structural characterization via X-ray diffraction.............................. 2

Powder X-ray diffraction patterns…………………………………………… 3

TGA………………………………………………………………………………………. 5

Structural characterization via X-ray diffraction

Table S1. Crystal data and details of measurements for the compounds described in this work (HKA = kojic acid; KA = kojiate anion).

**Compound**	**$Zn(KA)_2$**	**$Cu(KA)_2$[a]**	**$[Ag(HKA)(NO_3)]\cdot H_2O$**	**$[Ga(KA)_2(OH_2)_2][NO_3]\cdot H_2O$**
Chemical formula	$C_{12}H_{10}O_8Zn$	$C_{12}H_{10}CuO_8$	$C_6H_8AgNO_7\cdot H_2O$	$C_{12}H_{15}GaNO_{14}$
Formula weight	347.57	345.00	330.00	466.97
Temperature (K)	298	298	223	298
Crystal system	Triclinic	Monoclinic	Orthorhombic	Monoclinic
Space group	*P*-1	$P2_1$/a	$Pn2_1$/a	$P2_1$/c
a (Å)	5.1557(5)	15.313(2)	14.4029(5)	11.4594(11)
b (Å)	7.7602(7)	6.805(2)	3.6487(2)	21.4272(16)
c (Å)	7.8702(7)	11.923(2)	17.9803(7)	7.0775(15)
α (°)	94.492(7)	90	90	90
β (°)	108.982(8)	110.158(3)	90	90.157(13)
γ (°)	97.130(7)	90	90	90
V (Å^3)	293.08(5)	1171(3)	944.90(7)	1737.8(4)
Z	1	4	4	4
F(000)	176.0	700.0	645.0	948.0
ρ_{calc}(g·cm^{-3})	1.969	1.961	2.320	1.785
Mμ(mm^{-1})	2.137	3.070	2.164	1.662
R_{wp}	---	0.0417	---	---
N_{reflns}, N_{par}	1339, 98	---	1634, 153	3187, 266
R1 [F_0^2, I>2σ(I)]	0.0375	---	0.0362	0.0847
wR2 (all data)	0.0770	---	0.0591	0.2038

[a] Structural solution from powder data.

Powder X-ray diffraction

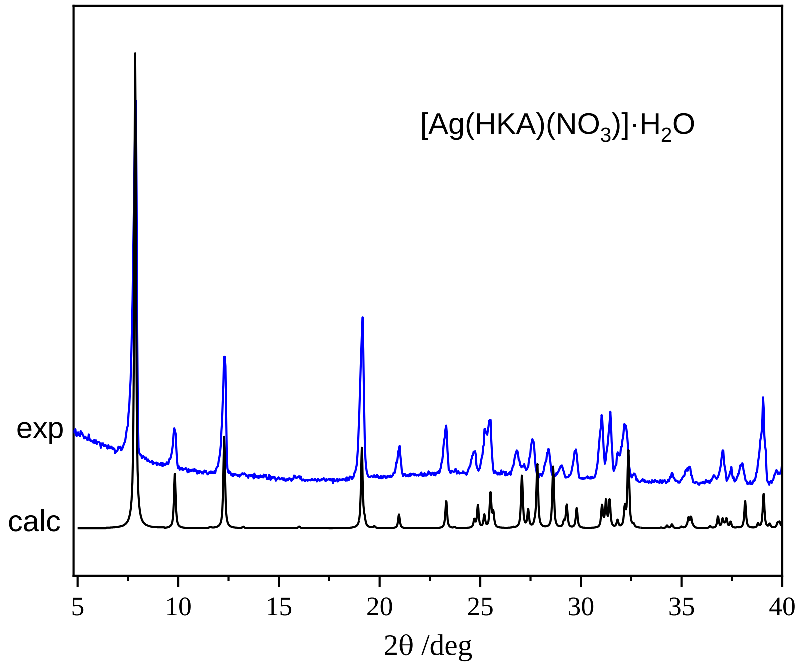

Figure S1: Comparison between the experimental pattern for the ball milling product of the reaction between HKA and $AgNO_3$ (blue line) and the pattern calculated from single crystal data for $[Ag(HKA)(NO_3)]\cdot H_2O$ (black line). Small differences in the peaks positions are due to the difference in the powder data collection temperature (RT) with respect to single crystal (223 K).

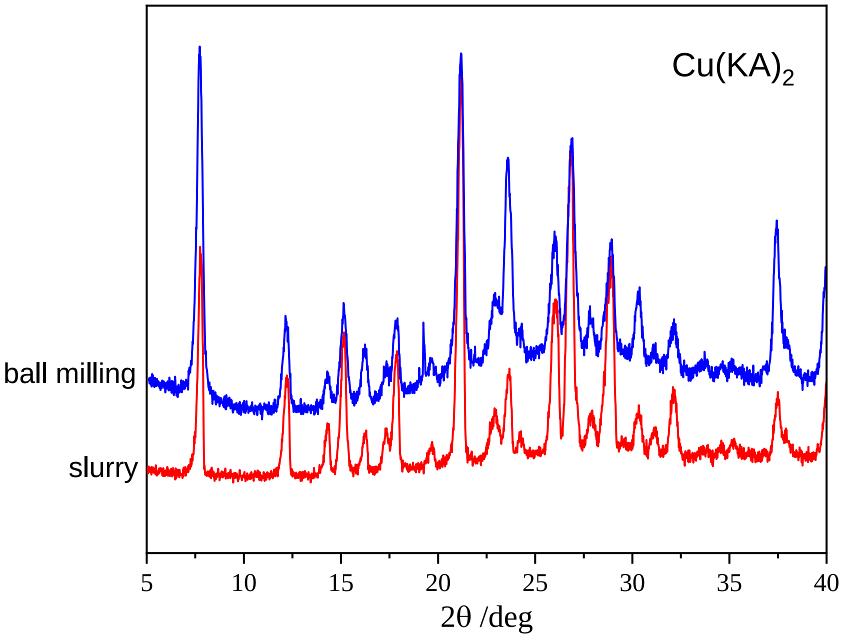

Figure S2: Comparison between XRD patterns for the slurry (red line) and ball milling (blue line) products of the reaction between HKA and $Cu(NO_3)_2$.

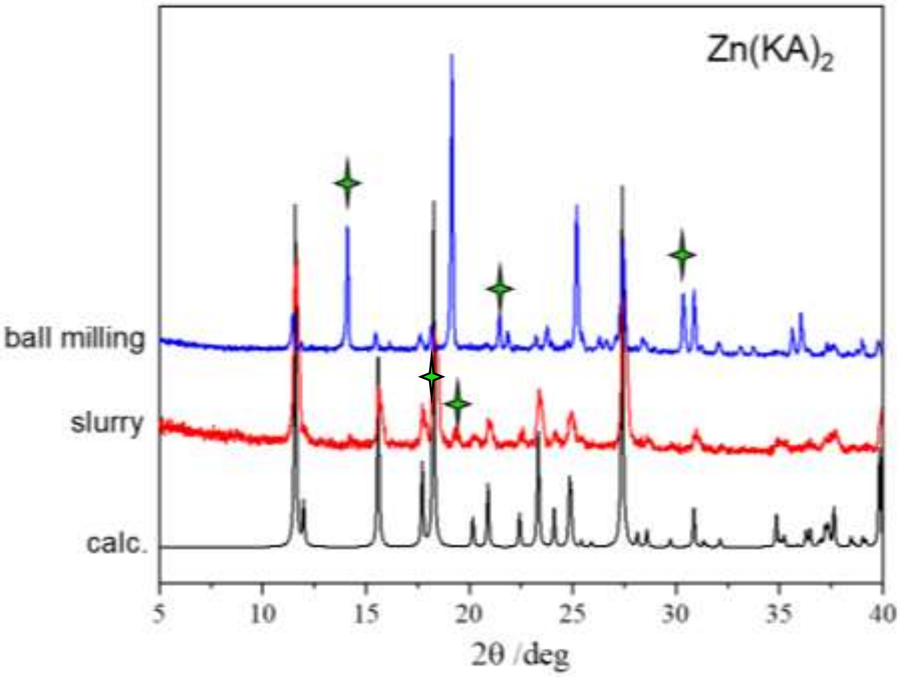

Figure S3: Comparison between the experimental patterns of ball milling (blue line) and slurry (red line) products of the reaction between HKA and $Zn(NO_3)_2$, and the pattern calculated from $Zn(KA)_2$ single crystal data (black line). Green stars indicate the presence of unreacted HKA (almost absent in the slurry product).

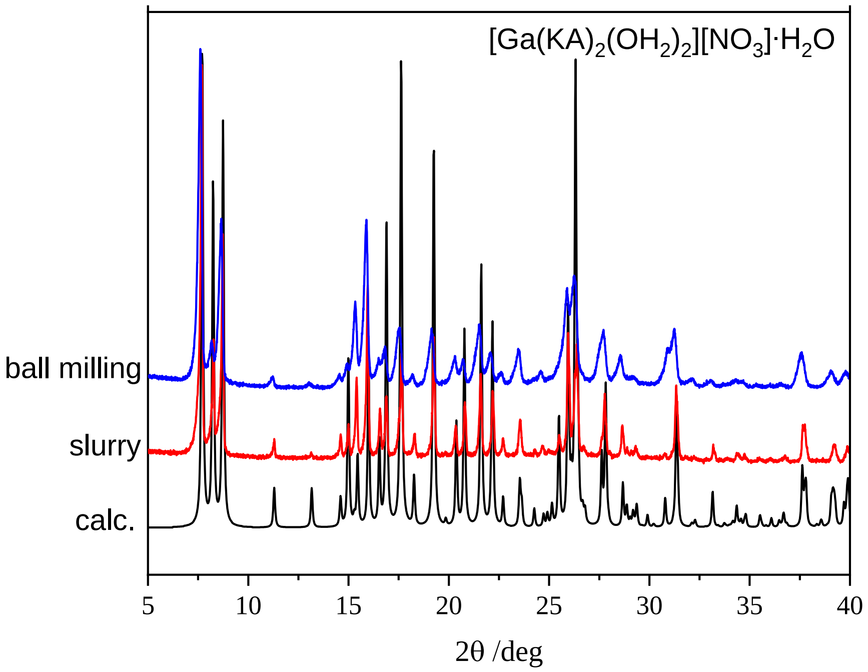

Figure S4: Comparison between the experimental patterns for the ball milling (blue line) and slurry (red line) products of the reaction between HKA and $Ga(NO_3)_3$, and the pattern calculated from single crystal data for $[Ga(KA)_2(OH_2)_2][NO_3]\cdot H_2O$ (black line).

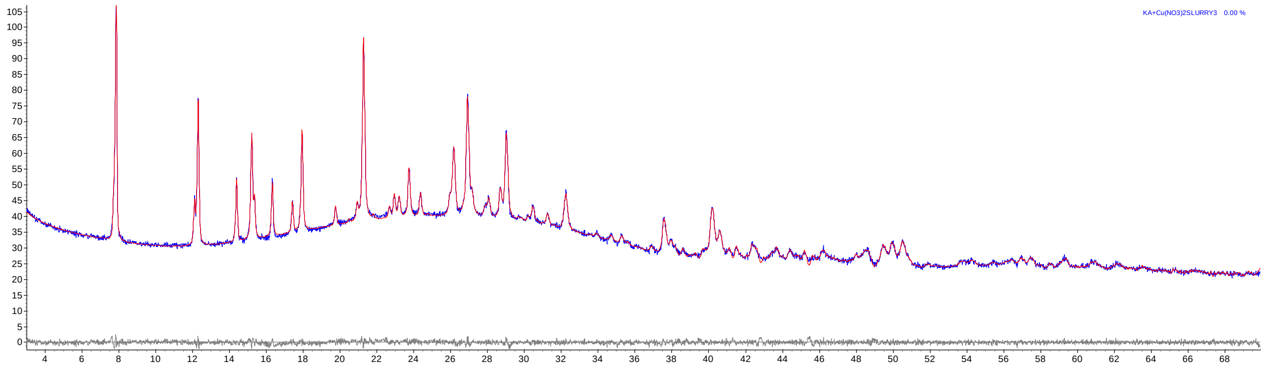

Figure S5: Pawley refinement plots for $Cu(KA)_2$.

Thermogravimetric analysis (TGA)

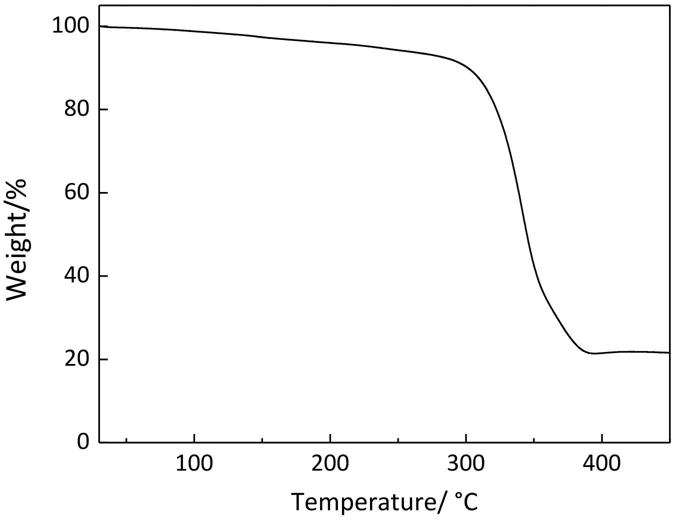

Figure S6: TGA trace for the product of the slurry reaction between HKA and $Cu(NO_3)_2$.
